# Neutrophil-related immune-inflammatory biomarkers influence the early progression of medial medullary infarction

**DOI:** 10.3389/fneur.2025.1528560

**Published:** 2025-02-26

**Authors:** Li-Min Li, Hao Cai

**Affiliations:** ^1^Department of Neurology, Tianjin Neurological Institute, Tianjin Medical University General Hospital, Tianjin, China; ^2^Department of Neurology, Tianjin Huanhu Hospital, Tianjin, China

**Keywords:** medial medullary infarction, inflammation, neutrophil-to-lymphocyte ratio, neutrophil-to-high-density lipoprotein cholesterol ratio, stroke

## Abstract

**Background:**

Medial medullary infarction (MMI) is a rare type of posterior circulation stroke for which accurate prognostication remains a challenge because of the limited predictive ability of the current models. Blood-derived biomarkers may provide valuable insights that extend beyond established prognostic factors. The aim of this study was to identify rapid and accessible biomarkers for predicting the early progression of MMI.

**Methods:**

Seventy patients with MMI and 83 sex- and age-matched healthy controls (HCs) were recruited for this study. Among them, 20 patients were allocated to the early progression cohort, and 50 patients were assigned to the nonprogression cohort. The laboratory blood indices were subsequently compared across these different cohorts. Receiver operating characteristic (ROC) curves were used to evaluate the predictive values of blood-derived indicators.

**Results:**

The white blood cell (WBC) count, neutrophil count, monocyte count, low-density lipoprotein cholesterol (LDL-C) level, total cholesterol (TC) concentration, WBC-to-high-density lipoprotein cholesterol (HDL-C) ratio (WHR), neutrophil-to-HDL-C ratio (NHR), monocyte-to-HDL-C ratio (MHR), monocyte-to-lymphocyte ratio (MLR), neutrophil-to-lymphocyte ratio (NLR) and platelet-to-lymphocyte ratio (PLR) were significantly greater in patients with MMI than in HCs (*p* < 0.05). The WBC count, neutrophil count, monocyte count, WHR, NHR, MHR, MLR, NLR and PLR were markedly higher in MMI patients with progression than in MMI patients without progression (*p* < 0.05). ROC curve analysis revealed that the WBC count, neutrophil count, monocyte count, MLR, NLR, PLR, NHR, and WHR were significant predictors of early progression. However, among these factors, the WBC count (AUC = 0.854*, p* < 0.001), neutrophil count (AUC = 0.878, *p* < 0.001), NLR (AUC = 0.861, *p* < 0.001), and NHR (AUC = 0.848, *p* < 0.001) had the highest levels of accuracy for predicting early progression in patients with MMI.

**Conclusion:**

The efficacy of the WBC count, neutrophil count, NLR and NHR is superior in predicting progression in patients with MMI. The current findings suggest that these indicators may serve as reliable, cost-effective, and innovative prognostic markers for MMI.

## 1 Introduction

Acute ischaemic stroke (AIS) is a severe neurologic disorder caused by disruption of cerebral perfusion, resulting in focal or global neurological impairment (1). AIS is the second most common cause of death worldwide, a major cause of adult disability and, thus, an increasing threat to global health (2, 3). In 2017, stroke became the leading cause of death in China (4). Medial medullary infarction (MMI) is a rare type of posterior circulation stroke that may cause rapid deterioration of neurological deficits and death. Even with diffusion-weighted imaging (DWI), approximately one-third of MMIs have false-negative results within the first 24 h after symptom onset. In addition, MMI does not always present with any unique symptoms and instead manifests as a common lacunar syndrome (5). Consequently, patients with MMI are often misdiagnosed with capsular/pontine lacunar stroke or other neurological disorders without repeated DWI examinations (5). Therefore, the early risk stratification and management of patients with MMI are crucial for improving their prognosis.

The inflammatory response is a key element in the pathophysiological mechanisms of AIS (6). After an ischaemic event, the infiltration of immune cells and the secretion of proinflammatory cytokines contribute to the secondary progression of neuronal damage, thereby worsening the blood–brain barrier disruption and brain oedema and increasing infarct volume (7). The total white blood cell count has been reported to be an independent predictor of stroke severity, degree of disability and mortality (8). White blood cell subtypes play crucial roles in the pathogenesis of atherogenesis and atherothrombosis, which are closely related to adverse cardiovascular and cerebrovascular events (9, 10). Recent investigations have demonstrated that an elevated neutrophil level is a negative prognostic indicator for patients with AIS, suggesting that immunomodulatory therapies may represent a promising treatment strategy for this condition (11). In contrast, significant decreases in lymphocyte counts have been observed in patients with adverse outcomes, underscoring the detrimental role of low lymphocyte counts in long-term functional recovery after AIS (12). Furthermore, monocytes are critical component of the poststroke inflammatory response; their numbers increase in the peripheral circulation following stroke onset, and they subsequently migrate to the infarcted region, thereby exacerbating brain injury (13).

Recently, the neutrophil-to-lymphocyte ratio (NLR) and monocyte-to-lymphocyte ratio (MLR) have been suggested to be novel, inexpensive, and repeatable markers of the systemic inflammatory response that are more suitable for routine use than traditional inflammatory markers such as tumor necrosis factor-alpha (TNF-*α*) and interleukin-6 (IL-6). These ratios can be easily calculated from a white blood cell (WBC) assay and determined under simple laboratory conditions (14–17). Some inflammatory indicators, such as the monocyte-to-high-density lipoprotein cholesterol ratio (MHR) and the neutrophil-to-high-density lipoprotein cholesterol ratio (NHR), have also been identified through the study of atherosclerotic cardiovascular disease (18, 19).

However, the relationships between these new inflammatory markers and disease severity and clinical outcomes in patients with MMI have not been fully elucidated. Therefore, the aim of this study was to investigate the predictive relationship between these new inflammatory markers and MMI.

## 2 Materials and methods

### 2.1 Study population

**Figure 1:**
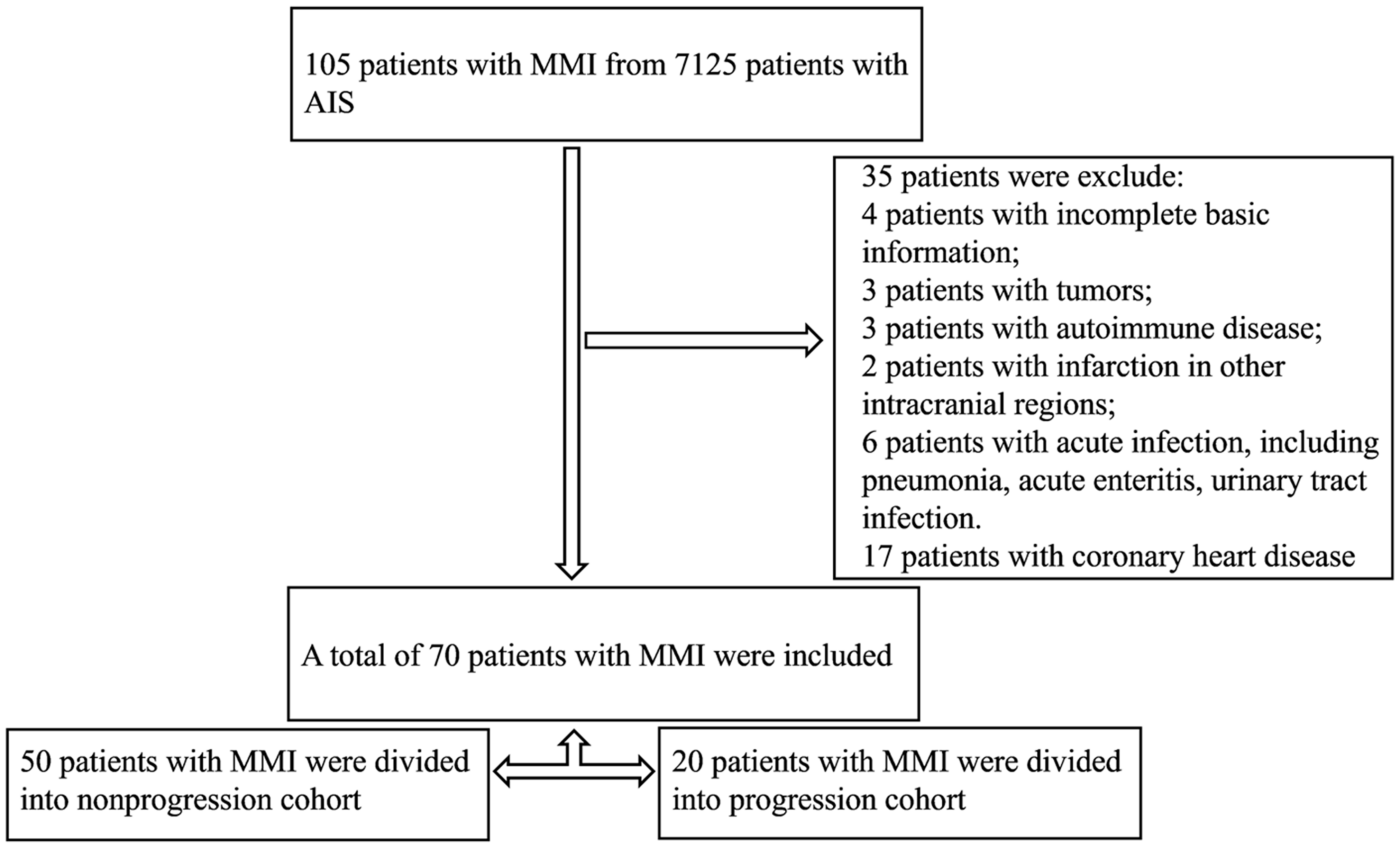
Study flow chart of participant inclusion.

The patients with AIS included in this retrospective cohort study were enrolled in the Neurology Department of Tianjin Medical University General Hospital from January 2020 to February 2024. Among the 7,125 patients diagnosed with AIS, 105 were identified with MMI. Of these, 70 met the criteria for inclusion into the study (Figure 1). The inclusion criteria were as follows: (1) hospitalization within 48 h of MMI onset; (2) acute ischaemic lesions of the medial medulla oblongata on DWI; (3) no myocardial infarction or stroke in the last year; and (4) a modified Rankin scale score of 0 before admission. In total, 83 healthy controls (HCs), matched for sex and age, were recruited from our health examination centre. The exclusion criteria were as follows: (1) acute infarction at other sites; (2) the presence of infectious disease, blood disease, liver or kidney function abnormalities, tumor, or autoimmune disease; (3) coronary heart disease; (4) age less than 18 years; or (5) incomplete clinical data. This study was approved by the Medical Research Ethics Committee at Tianjin Medical University General Hospital. All participants provided written informed consent in accordance with the Declaration of Helsinki.

### 2.2 Data collection

Demographic and clinical information, including age, sex, and history of hypertension, diabetes, atrial fibrillation, hyperlipidaemia, smoking, and alcohol consumption, was collected. On admission, all patients underwent physical examination and imaging examinations, including brain magnetic resonance imaging (MRI), brain magnetic resonance angiography (MRA) and computed tomography angiography (CTA). Blood samples were collected 24 h after admission. The MLR, NLR, and platelet-to-lymphocyte ratio (PLR) were defined by the monocyte, neutrophil, and platelet-to-lymphocyte counts, respectively. The WBC-to-high-density lipoprotein cholesterol (HDL-C) ratio (WHR), NHR, and MHR were separately determined by calculating the WBC, neutrophil, and monocyte counts relative to the HDL-C level. The National Institutes of Health Stroke Scale (NIHSS) was used to assess the severity of nervous system damage as evaluated by trained neurologists. Early progression was defined as an increase in the NIHSS score by ≥1 point in motor strength or ≥ 2 points in the total score within the first week after symptom onset (20). Therefore, the patients were divided into an early progression group and a nonprogression group.

### 2.3 Statistical analysis

The data were analyzed via SPSS 26.0. The normality of all variables was assessed by means of the Kolmogorov–Smirnov test. Descriptive statistics such as the mean and standard deviation are reported herein for continuous variables, and the frequency (%) is presented for categorical variables. Student’s t test and the Mann–Whitney U test were used for between-group comparisons of continuous variables, and the chi–square test was used for comparisons of categorical variables. The predictive ability of the inflammatory biomarkers was evaluated by means of the area under the receiver operating characteristic (ROC) curve. The Delong test was used to evaluate differences between two areas under the curve (AUCs). A *p* value of <0.05 indicated statistical significance.

## 3 Results

### 3.1 Characteristics of the research subjects

Among the 70 patients diagnosed with MMI, 31 (44.29%) were identified as smokers, 14 (20%) reported a history of alcohol consumption, 53 (75.71%) had been diagnosed with hypertension, 34 (48.57%) presented with hyperlipidaemia, and 22 (31.43%) were diagnosed with diabetes mellitus (DM). Notably, no patients exhibited atrial fibrillation (AF). Additionally, three patients (4.28%) received intravenous thrombolysis. Furthermore, there was no significant difference in age, sex, medical history, or NIHSS score at admission between MMI patients with progression and those without progression (Table 1).

**Table 1:** Demographic characteristics and clinical data of MMI patients.

	Nonprogression (*N* = 50)	Progression (*N* = 20)	*p* value
Sex, F/M	21/29	7/13	0.589
Age, *y*	61.24 ± 10.99	63.55 ± 14.85	0.476
Medical history, *n* (%)			
Smoking	21 (42.00)	10 (50.00)	0.543
Drinking	9 (18.00)	5(25.00)	0.508
‌Hypertension	36 (72.00)	17 (85.00)	0.252
DM	13 (26.00)	9 (45.00)	0.122
Atrial fibrillation	0	0	1.000
Hyperlipidaemia	23 (46.00)	11 (55.00)	0.496
ITT	2(4.00)	1 (5.00)	0.852
NIHSS at admission	4.66 ± 2.49	5.05 ± 2.19	0.542
NIHSS at nadir	4.66 ± 2.49	7.85 ± 2.48	< 0.001

MMI, medial medullary infarction; N, number; F, female; M, male; y, year; DM, diabetes mellitus; ITT, intravenous thrombolytic therapy; NIHSS, National Institutes of Health Stroke Scale.

### 3.2 Comparison of inflammation-related biomarkers between MMI patients and HCs

The WBC count (*p* < 0.001), neutrophil count (*p* < 0.001), monocyte count (*p* = 0.015), low-density lipoprotein cholesterol (LDL-C) level (*p* < 0.001), and total cholesterol (TC) level (*p* = 0.007) were significantly higher in patients with MMI than in HCs. The lymphocyte count (*p* = 0.048) in patients with MMI was significantly lower than that in HCs. In addition, there were significant increases in the WHR (*p* < 0.001), NHR (*p* < 0.001), MHR (*p* = 0.033), MLR (*p* < 0.001), NLR (*p* < 0.001), and PLR (*p* = 0.013) in patients with MMI compared with HCs. There were no significant differences in the PLT counts, HDL-C levels or triglyceride (TG) concentrations between patients with MMI and HCs (*p* > 0.05) (Table 2).

**Table 2:** Comparison of inflammation-related biomarkers between patients with MMI and HCs.

	MMI (*N* = 70)	HCs (*N* = 83)	*P* value
WBCs (×10^9^/L)	8.93 ± 3.68	6.22 ± 1.27	< 0.001
Neutrophils (×10^9^/L)	6.67 ± 3.63	3.44 ± 1.03	< 0.001
Monocytes (×10^9^/L)	0.49 ± 0.23	0.42 ± 0.12	0.015
Lymphocytes (×10^9^/L)	1.78 ± 1.51	2.16 ± 0.69	0.048
PLTs	234.13 ± 62.70	228.92 ± 56.65	0.590
HDL-C (mmol/L)	1.18 ± 0.30	1.16 ± 0.28	0.704
LDL-C (mmol/L)	3.29 ± 0.78	2.83 ± 0.83	< 0.001
TG (mmol/L)	1.59 ± 0.61	1.48 ± 0.60	0.272
TC (mmol/L)	5.14 ± 1.14	4.64 ± 1.08	0.007
WHR	7.96 ± 3.77	5.59 ± 1.49	< 0.001
NHR	5.88 ± 3.37	3.07 ± 1.03	< 0.001
MHR	0.44 ± 0.25	0.37 ± 0.13	0.033
MLR	0.35 ± 0.26	0.21 ± 0.11	< 0.001
NLR	5.28 ± 5.16	1.86 ± 1.46	< 0.001
PLR	165.56 ± 93.29	123.92 ± 109.86	0.013

MMI, medial medullary infarction; HCs, healthy controls; *N*, number; WBCs, white blood cells; PLTs, blood platelets; HDL-C, high-density lipoprotein cholesterol; LDL-C, low-density lipoprotein cholesterol; TG, triglyceride; TC, total cholesterol; WHR, WBC-to-HDL-C ratio; NHR, neutrophil-to-HDL-C ratio; MHR, monocyte-to-HDL-C ratio; MLR, monocyte-to-lymphocyte ratio; NLR, neutrophil-to-lymphocyte ratio; PLR, blood platelet-to-lymphocyte ratio.

### 3.3 Comparison of inflammation-related biomarkers between MMI patients with and without progression

The WBC count (*p* < 0.001), neutrophil count (*p* < 0.001), monocyte count (*p* = 0.002), WHR (*p* < 0.001), NHR (*p* < 0.001), MHR (*p* = 0.035), MLR (*p* < 0.001), NLR (*p* < 0.001) and PLR (*p* = 0.007) were markedly higher in MMI patients with progression than in MMI patients without progression. There were no significant differences in the lymphocyte count, PLT count or HDL-C, LDL-C, TG or TC levels between the groups (*p* > 0.05) (Table 3).

**Table 3:** Comparison of inflammation-related biomarkers between MMI patients with and without progression.

	Nonprogression (*N* = 50)	Progression (*N* = 20)	*P* value
WBCs (×10^9^/L)	7.58 ± 2.47	12.28 ± 4.13	< 0.001
Neutrophils (×10^9^/L)	5.24 ± 2.21	10.24 ± 4.09	< 0.001
Monocytes (×10^9^/L)	0.44 ± 0.16	0.62 ± 0.30	0.002
Lymphocytes (×10^9^/L)	1.99 ± 1.71	1.27 ± 0.57	0.069
PLTs (×10^9^/L)	231.48 ± 64.51	240.75 ± 58.99	0.580
HDL-C (mmol/L)	1.15 ± 0.20	1.23 ± 0.48	0.338
LDL-C (mmol/L)	3.31 ± 0.72	3.25 ± 0.92	0.779
TG (mmol/L)	1.63 ± 0.62	1.50 ± 0.62	0.421
TC (mmol/L)	5.14 ± 1.02	5.14 ± 1.43	0.993
WHR	6.68 ± 2.12	11.17 ± 4.99	< 0.001
NHR	4.59 ± 1.75	9.14 ± 4.20	< 0.001
MHR	0.39 ± 0.14	0.59 ± 0.39	0.035
MLR	0.26 ± 0.13	0.58 ± 0.34	< 0.001
NLR	3.20 ± 1.60	10.49 ± 7.01	< 0.001
PLR	140.71 ± 61.99	227.70 ± 126.53	0.007

MMI: Medial medullary infarction; N, number; WBCs, white blood cells; PLTs, blood platelets; HDL-C, high-density lipoprotein cholesterol; LDL-C, low-density lipoprotein cholesterol; TG, triglyceride; TC, total cholesterol; WHR, WBC-to-HDL-C ratio; NHR, neutrophil-to-HDL-C ratio; MHR, monocyte-to-HDL-C ratio; MLR, monocyte-to-lymphocyte ratio; NLR, neutrophil-to-lymphocyte ratio; PLR, blood platelet-to-lymphocyte ratio.

### 3.4 Receiver operating characteristic (ROC) curve analysis for the ability of biomarkers to predict early progression of MMI

**Figure 2:**
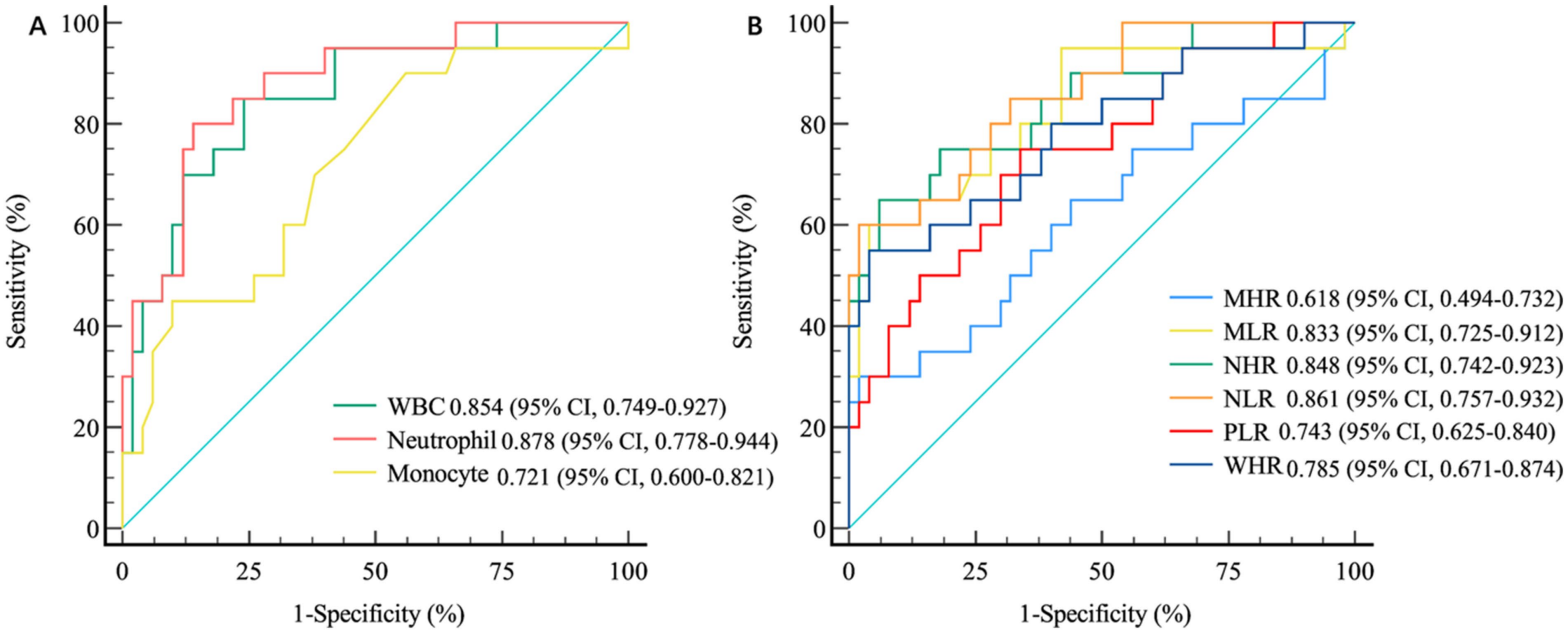
The differential accuracy of inflammation-related biomarkers in MMI patients with and without progression. **(A)** ROC Curve Analysis of White Blood Cells and Their Subtypes. **(B)** ROC Curve Analysis of Inflammation-Related Ratio Indicators. MMI: Medial medullary infarction; WBC, white blood cell; WHR, WBC-to-high-density lipoprotein cholesterol ratio; NHR, neutrophil-to-high-density lipoprotein cholesterol ratio; MHR, monocyte-to-high-density lipoprotein cholesterol ratio; MLR, monocyte-to-lymphocyte ratio; NLR, neutrophil-to-lymphocyte ratio; PLR, blood platelet-to-lymphocyte ratio; ROC, receiver operating characteristic.

The ROC curves revealed that the WBC count (AUC = 0.854, 95% CI 0.749–0.927, *p* < 0.001), neutrophil count (AUC = 0.878, 95% CI 0.778–0.944, *p* < 0.001), and monocyte count (AUC = 0.721, 95% CI 0.600–0.821, *p* < 0.001) were significant indicators of early prognosis in patients with MMI. The performance of both the WBC count (AUC = 0.854*, p* < 0.001) and neutrophil count (AUC = 0.878, *p* < 0.001) was superior for predicting early progression in patients with MMI. However, no significant differences in their predictive efficiencies were observed (DeLong’s test*, p* = 0.084) (Figure 2A). Furthermore, the AUC of the NLR and NHR were 0.861 (95% CI 0.757–0.932, *p* < 0.001) and 0.848 (95% CI 0.742–0.923, *p* < 0.001), respectively. While, the AUCs of the MLR, WHR and PLR were 0.833 (95% CI 0.725–0.912, *p* < 0.001), 0.785 (95% CI 0.671–0.874, *p* < 0.001) and 0.743 (95% CI 0.625–0.840, *p* < 0.001), respectively. In evaluating the efficacy of the NHR, NLR, MLR, WHR, and PLR in differentiating between MMI patients with progression and those without progression, the highest AUCs were achieved for both the NHR (AUC = 0.848, *p* < 0.001) and the NLR (AUC = 0.861, *p* < 0.001). DeLong’s test demonstrated that the NHR significantly outperformed the WHR (*p* = 0.014), whereas the NLR surpassed the PLR (*p* = 0.007) in predicting early progression in patients with MMI (Figure 2B).

## 4 Discussion

In this study, the correlation between clinically significant changes in biomarkers comprising routine blood and biochemical indices and the prognosis of patients with MMI was investigated. The main findings of this investigation were as follows: (1) WBC count, neutrophil count, monocyte count, LDL-C level, TC level, WHR, NHR, MHR, MLR, NLR and PLR were significantly higher in patients with MMI than in HCs. However, the lymphocyte count in patients with MMI was significantly lower than that in HCs (2). The WBC count, neutrophil count, monocyte count, WHR, NHR, MHR, MLR, NLR and PLR were markedly higher in MMI patients with progression compared with those without progression (3). ROC curve analysis revealed that the WBC count, neutrophil count, monocyte count, MLR, NLR, PLR, NHR and WHR are significant predictors of early disease progression. Furthermore, the performance of the WBC and neutrophil counts for predicting early progression in patients with MMI was superior among the routine biochemical indicators. However, there were no significant differences in predictive efficiency between these two indicators. The NHR and NLR outperformed the MLR, PLR and WHR in the prediction of poor outcomes in MMI.

MMI is a rare type of ischaemic stroke that accounts for approximately 0.5–1.5% of all ischaemic strokes and manifests with diverse neurological symptoms. MMI is characterized by a high rate of progression and poor functional prognosis (5, 21). Nevertheless, the prediction of cerebral infarction remains a notable obstacle due to the limited predictive ability of the current models. Therefore, the present study was conducted to develop an innovative approach for prognostic estimation, with the objective of identifying a direct and effective index that would enhance the prognostic assessment of patients with MMI. The poststroke inflammatory response is widely believed to be associated with infarction progression (22). Additionally, atherosclerosis is characterized by chronic and persistent inflammation of the arteries, which is marked by immune cell infiltration (23). As key contributors to the inflammatory response, different subtypes of white blood cells are thought to exacerbate the complex situation of secondary brain injury following a stroke.

Our study revealed that the WBC count, the monocyte count, and especially the neutrophil count were higher in patients with MMI than in HCs. However, the lymphocyte count in patients with MMI was significantly lower than that in HCs. The inflammatory response plays a crucial role in tissue damage caused by cerebral ischaemia (11). Inflammation is not only involved in the development of atherosclerosis, which is a key factor in the onset of cerebrovascular diseases but also enhances the local immune response at the site of injury, leading to brain cell death and worsening neurological dysfunction (24). Within hours after ischaemic stroke, the local inflammatory response can activate certain types of WBCs, particularly neutrophils. Neutrophils first migrate to the inflamed area within blood vessels and contribute to increased expression of inflammatory mediators and cell adhesion molecules, thereby directly or indirectly exacerbating damage in the affected area (25). Moreover, increased neutrophil counts may serve as a predictor of unfavorable outcomes in stroke patients (26), which aligns with the findings of the current study.

Monocytes also play crucial roles in the initiation and progression of atherosclerotic disease. They can migrate from the bloodstream to tissues when triggered by various signals, where they can transform into inflammatory dendritic cells and foam cells. Monocytes subsequently activate the production of proinflammatory cytokines, matrix metalloproteinases, and reactive oxygen species, all of which are integral to the initiation, development, and eventual rupture of atherosclerotic plaques (27, 28). Consequently, the observed increase in the number of circulating monocytes indicates that these cells surrounding atherosclerotic plaques have increased responsiveness to signaling cues, thereby exacerbating the inflammatory response, intensifying arterial stenosis, and increasing the risk of plaque rupture. A significant correlation between lymphocyte reduction and acute coronary syndrome has been established in a previous study (29). In the present study, we also observed lower lymphocyte counts in patients with MMI than in HCs. The mechanism behind these findings can be attributed to the increase in catecholamine and cortisol levels under stress, which leads to excessive lymphocyte apoptosis (30, 31). Therefore, an increase in monocyte levels and a reduction in lymphocyte counts might significantly contribute to the pathogenesis of MMI.

In the present study, we observed that the levels of blood lipids, including TC and, particularly, LDL-C, were significantly elevated in patients with MMI compared with HCs. However, there was no significant difference in LDL levels between patients with early progression and those without progression. It is widely accepted that increased LDL levels are critical contributors to atherosclerotic inflammation, indicating a positive correlation between the degree of inflammation in atherosclerosis and LDL-C levels (32). During the process of atherosclerosis, elevated LDL-C stimulates the secretion of inflammatory factors or triggers inflammatory responses through various mechanisms. Oxidized LDL-C is involved in the formation of substances such as costimulatory molecules (CD80, CD86, and CD40), proinflammatory prostaglandins, and proinflammatory cytokines (33, 34). Monocytes are recruited to areas of endothelial injury induced by LDL, and LDL plays a role in cell morphology and viability during the early stage of the transdifferentiation of monocytes into macrophages (35). Consequently, LDL is a significant contributor to the pathogenesis of MMI. However, it has limited specificity in predicting the progression of MMI.

In this study, the WHR, NHR, MHR, MLR, NLR, and PLR were significantly greater in patients with MMI than in HCs. While similar trends were noted in patients with early progression of MMI compared with patients without progression, ROC curve analysis revealed that among these indicators, the predictive performance of NHR and NLR for the early progression of MMI were superior among these indicators. The NHR and NLR are potential indices of inflammation and are readily available. There are numerous reasons why NHR levels are elevated during MMI. First, neutrophils, which constitute the main subgroup of WBCs, respond to early infections after stroke (36). Many activated neutrophils can influence the composition and function of HDL-C by altering the structure and content of various apolipoproteins. For example, they can degrade apolipoprotein E, apolipoprotein a-I and apolipoprotein a-II to reduce the reverse transport function of cholesterol, thereby resulting in an increase in the NHR in acute cerebral infarction (37–39). Second, in animal models, HDL-C, as the principal component protein, might interfere with the release of neutrophils by restraining the synthesis of cytokines such as IL-6 and IL-1β (40) and enhance the clearance efficacy of neutrophils in inflammatory tissues by reducing the expression of granulocyte colony stimulating factor (G-CSF) to suppress the inflammatory response mediated by neutrophils. G-CSF is a crucial regulatory factor for the generation and survival of neutrophils (41, 42). In summary, the reduced inhibitory impact of HDL-C on neutrophil development, differentiation, and adhesion leads to an increased NHR in patients with MMI. While the aetiology of an increased NLR in patients with MMI is likely multifactorial, the primary and most influential factor is the acute stress response or hypercortisolism associated with stroke, leading to a lymphopenia–neutrophilia reaction (43). Additionally, NLR elevation may be attributable to stroke-induced immune depression resulting in lymphopenia and early infection leading to neutrophilia. The assessment and monitoring of the NLR could prove valuable as a clinical parameter for stroke patients. Furthermore, the NLR and the NHR have been well established as independent risk factors for poor short-term prognosis in patients with AIS (44). In particular, Zhao et al. demonstrated that the NHR (AUC = 0.689) and the NLR (AUC = 0.723) might serve as significant predictors of early progression in patients with anterior choroidal artery (AChA) infarction (45). Notably, our findings revealed the enhanced predictive performance of NHR and NLR in patients with MMI, with AUCs of 0.848 and 0.861, respectively. The reasons for these differences in predictive efficacy between AChA infarction and MMI remain unclear. Future research should focus on systematic comparative analyses of these inflammatory biomarkers across different AIS subtypes, which could provide valuable insights into distinct pathophysiological processes and potentially guide the development of subtype-specific therapeutic strategies for improved patient outcomes.

This study has several limitations. First, this study has a limited sample size. Thus, larger cohorts from multicentre trials and further prospective follow-up studies are warranted. Second, this investigation was observational in nature and lacked any interventions. Consequently, the potential benefits of anti-inflammatory therapy for patients with MMI remain unclear, so large-scale prospective interventional studies involving patients with MMI are imperative. Third, owing to the cross-sectional nature of the data collection in this study, changes over time could not be tracked. Future research should incorporate longitudinal approaches to capture the dynamic changes in the levels of biomarkers and their correlations with clinical outcomes. Finally, in this study, only a small number of patients who received intravenous thrombolysis in the early stages of MMI were included. In future studies, larger cohorts with more patients receiving thrombolysis should be included to better understand the impact of thrombolysis on disease progression and biomarker profiles.

Our findings suggest that high WBC, neutrophil, monocyte, WHR, NHR, MHR, MLR, NLR and PLR values in patients with MMI are associated with a poor early prognosis. Moreover, the performance of the WBC count, neutrophil count, NLR and NHR in predicting the progression of patients with MMI is superior among the biomarkers evaluated. When early WBC counts, neutrophil counts, NLR and NHR are high, clinicians should pay more attention to the condition of the patients with MMI to improve the disease prognosis.

## Data Availability

The original contributions presented in the study are included in the article/supplementary material, further inquiries can be directed to the corresponding author/s.

## References

[1] Loh HC Lim R Lee KW Ooi CY Chuan DR Looi I. Effects of vitamin E on stroke: a systematic review with meta-analysis and trial sequential analysis. Stroke Vasc Neurol. (2021) 6:109–20. doi: 10.1136/svn-2020-000519, PMID: 33109618 PMC8005911

[2] Mathers CD Loncar D. Projections of global mortality and burden of disease from 2002 to 2030. PLoS Med. (2006) 3:e442. doi: 10.1371/journal.pmed.0030442, PMID: 17132052 PMC1664601

[3] Johnston SC Mendis S Mathers CD. Global variation in stroke burden and mortality: estimates from monitoring, surveillance, and modelling. Lancet Neurol. (2009) 8:345–54. doi: 10.1016/S1474-4422(09)70023-7, PMID: 19233730

[4] Zhou M Wang H Zeng X Yin P Zhu J Chen W. Mortality, morbidity, and risk factors in China and its provinces, 1990-2017: a systematic analysis for the global burden of disease study 2017. Lancet. (2019) 394:1145–58. doi: 10.1016/S0140-6736(19)30427-1, PMID: 31248666 PMC6891889

[5] Shono Y Koga M Toyoda K Matsuoka H Yokota C Uehara T. Medial medullary infarction identified by diffusion-weighted magnetic resonance imaging. Cerebrovasc Dis. (2010) 30:519–24. doi: 10.1159/000319887, PMID: 20861624

[6] Kim JY Kawabori M Yenari MA. Innate inflammatory responses in stroke: mechanisms and potential therapeutic targets. Curr Med Chem. (2014) 21:2076–97. doi: 10.2174/0929867321666131228205146, PMID: 24372209 PMC4104826

[7] Petrovic-Djergovic D Goonewardena SN Pinsky DJ. Inflammatory disequilibrium in stroke. Circ Res. (2016) 119:142–58. doi: 10.1161/CIRCRESAHA.116.308022, PMID: 27340273 PMC5138050

[8] Furlan JC Vergouwen MD Fang J Silver FL. White blood cell count is an independent predictor of outcomes after acute ischaemic stroke. Eur J Neurol. (2014) 21:215–22. doi: 10.1111/ene.12233, PMID: 23848934

[9] Kounis NG Soufras GD Tsigkas G Hahalis G. White blood cell counts, leukocyte ratios, and eosinophils as inflammatory markers in patients with coronary artery disease. Clin Appl Thromb Hemost. (2015) 21:139–43. doi: 10.1177/1076029614531449, PMID: 24770327

[10] Yamamoto E Sugiyama S Hirata Y Tokitsu T Tabata N Fujisue K. Prognostic significance of circulating leukocyte subtype counts in patients with coronary artery disease. Atherosclerosis. (2016) 255:210–6. doi: 10.1016/j.atherosclerosis.2016.08.033, PMID: 27612676

[11] Maestrini I Strbian D Gautier S Haapaniemi E Moulin S Sairanen T. Higher neutrophil counts before thrombolysis for cerebral ischemia predict worse outcomes. Neurology. (2015) 85:1408–16. doi: 10.1212/WNL.0000000000002029, PMID: 26362283 PMC4626239

[12] Kim J Song TJ Park JH Lee HS Nam CM Nam HS. Different prognostic value of white blood cell subtypes in patients with acute cerebral infarction. Atherosclerosis. (2012) 222:464–7. doi: 10.1016/j.atherosclerosis.2012.02.042, PMID: 22460048

[13] Kaito M Araya S Gondo Y Fujita M Minato N Nakanishi M. Relevance of distinct monocyte subsets to clinical course of ischemic stroke patients. PLoS One. (2013) 8:e69409. doi: 10.1371/journal.pone.0069409, PMID: 23936327 PMC3732285

[14] Ji H Li Y Fan Z Zuo B Jian X Li L. Monocyte/lymphocyte ratio predicts the severity of coronary artery disease: a syntax score assessment. BMC Cardiovasc Disord. (2017) 17:90. doi: 10.1186/s12872-017-0507-4, PMID: 28359298 PMC5374608

[15] Zahorec R. Ratio of neutrophil to lymphocyte counts--rapid and simple parameter of systemic inflammation and stress in critically ill. Bratisl Lek Listy. (2001) 102:5–14. PMID: 11723675

[16] Balta S Demirkol S Unlu M Arslan Z Celik T. Neutrophil to lymphocyte ratio may be predict of mortality in all conditions. Br J Cancer. (2013) 109:3125–6. doi: 10.1038/bjc.2013.598, PMID: 24084765 PMC3859933

[17] Balta S Demirkol S Celik T Kucuk U Unlu M Arslan Z. Association between coronary artery ectasia and neutrophil-lymphocyte ratio. Angiology. (2013) 64:627–32. doi: 10.1177/0003319713480424, PMID: 23471489

[18] He Y Kothari V Bornfeldt KE. High-density lipoprotein function in cardiovascular disease and diabetes mellitus. Arterioscler Thromb Vasc Biol. (2018) 38:e10–6. doi: 10.1161/ATVBAHA.117.310222, PMID: 29367232 PMC5804739

[19] Wen Y Zhan X Wang N Peng F Feng X Wu X. Monocyte/lymphocyte ratio and cardiovascular disease mortality in peritoneal Dialysis patients. Mediat Inflamm. (2020) 2020:9852507–9. doi: 10.1155/2020/9852507, PMID: 32214908 PMC7048939

[20] Li H Dai Y Wu H Luo L Wei L Zhou L. Predictors of early neurologic deterioration in acute pontine infarction. Stroke. (2020) 51:637–40. doi: 10.1161/STROKEAHA.119.027239, PMID: 31795900

[21] Toyoda K Imamura T Saku Y Oita J Ibayashi S Minematsu K. Medial medullary infarction: analyses of eleven patients. Neurology. (1996) 47:1141–7. doi: 10.1212/wnl.47.5.1141, PMID: 8909419

[22] Lambertsen KL Biber K Finsen B. Inflammatory cytokines in experimental and human stroke. J Cereb Blood Flow Metab. (2012) 32:1677–98. doi: 10.1038/jcbfm.2012.88, PMID: 22739623 PMC3434626

[23] Medina-Leyte DJ Zepeda-Garcia O Dominguez-Perez M Gonzalez-Garrido A Villarreal-Molina T Jacobo-Albavera L. Endothelial dysfunction, inflammation and coronary artery disease: potential biomarkers and promising Therapeutical approaches. Int J Mol Sci. (2021) 22:22. doi: 10.3390/ijms22083850, PMID: 33917744 PMC8068178

[24] Garcia-Bonilla L Moore JM Racchumi G Zhou P Butler JM Iadecola C. Inducible nitric oxide synthase in neutrophils and endothelium contributes to ischemic brain injury in mice. J Immunol. (2014) 193:2531–7. doi: 10.4049/jimmunol.1400918, PMID: 25038255 PMC4147670

[25] Enzmann G Mysiorek C Gorina R Cheng YJ Ghavampour S Hannocks MJ. The neurovascular unit as a selective barrier to polymorphonuclear granulocyte (PMN) infiltration into the brain after ischemic injury. Acta Neuropathol. (2013) 125:395–412. doi: 10.1007/s00401-012-1076-3, PMID: 23269317 PMC3578720

[26] Liu H Wang R Shi J Zhang Y Huang Z You S. Baseline neutrophil counts and neutrophil ratio may predict a poor clinical outcome in minor stroke patients with intravenous thrombolysis. J Stroke Cerebrovasc Dis. (2019) 28:104340. doi: 10.1016/j.jstrokecerebrovasdis.2019.104340, PMID: 31462383

[27] Woollard KJ Geissmann F. Monocytes in atherosclerosis: subsets and functions. Nat Rev Cardiol. (2010) 7:77–86. doi: 10.1038/nrcardio.2009.228, PMID: 20065951 PMC2813241

[28] Ley K Miller YI Hedrick CC. Monocyte and macrophage dynamics during atherogenesis. Arterioscler Thromb Vasc Biol. (2011) 31:1506–16. doi: 10.1161/ATVBAHA.110.221127, PMID: 21677293 PMC3133596

[29] Camici PG D'Amati G Rimoldi O. Coronary microvascular dysfunction: mechanisms and functional assessment. Nat Rev Cardiol. (2015) 12:48–62. doi: 10.1038/nrcardio.2014.160, PMID: 25311229

[30] Cioca DP Watanabe N Isobe M. Apoptosis of peripheral blood lymphocytes is induced by catecholamines. Jpn Heart J. (2000) 41:385–98. doi: 10.1536/jhj.41.385, PMID: 10987355

[31] Kruger K Agnischock S Lechtermann A Tiwari S Mishra M Pilat C. Intensive resistance exercise induces lymphocyte apoptosis via cortisol and glucocorticoid receptor-dependent pathways. J Appl Physiol. (2011) 110:1226–32. doi: 10.1152/japplphysiol.01295.2010 21393471

[32] Tunon J Badimon L Bochaton-Piallat ML Cariou B Daemen MJ Egido J. Identifying the anti-inflammatory response to lipid lowering therapy: a position paper from the working group on atherosclerosis and vascular biology of the European Society of Cardiology. Cardiovasc Res. (2019) 115:10–9. doi: 10.1093/cvr/cvy293, PMID: 30534957 PMC6302260

[33] Shapiro MD Fazio S. From lipids to inflammation: new approaches to reducing atherosclerotic risk. Circ Res. (2016) 118:732–49. doi: 10.1161/CIRCRESAHA.115.306471, PMID: 26892970

[34] Hansson GK Hermansson A. The immune system in atherosclerosis. Nat Immunol. (2011) 12:204–12. doi: 10.1038/ni.2001, PMID: 21321594

[35] Escate R Padro T Badimon L. LDL accelerates monocyte to macrophage differentiation: effects on adhesion and anoikis. Atherosclerosis. (2016) 246:177–86. doi: 10.1016/j.atherosclerosis.2016.01.002, PMID: 26800307

[36] Pektezel MY Yilmaz E Arsava EM Topcuoglu MA. Neutrophil-to-lymphocyte ratio and response to intravenous thrombolysis in patients with acute ischemic stroke. J Stroke Cerebrovasc Dis. (2019) 28:1853–9. doi: 10.1016/j.jstrokecerebrovasdis.2019.04.014, PMID: 31072698

[37] Bergt C Marsche G Panzenboeck U Heinecke JW Malle E Sattler W. Human neutrophils employ the myeloperoxidase/hydrogen peroxide/chloride system to oxidatively damage apolipoprotein A-I. Eur J Biochem. (2001) 268:3523–31. doi: 10.1046/j.1432-1327.2001.02253.x, PMID: 11422382

[38] Cogny A Atger V Paul JL Soni T Moatti N. High-density lipoprotein 3 physicochemical modifications induced by interaction with human polymorphonuclear leucocytes affect their ability to remove cholesterol from cells. Biochem J. (1996) 314:285–92. doi: 10.1042/bj3140285, PMID: 8660296 PMC1217038

[39] Cogny A Paul JL Atger V Soni T Moatti N. Structural changes of high-density-lipoprotein apolipoproteins following incubation with human polymorphonuclear cells. Eur J Biochem. (1994) 222:965–73. doi: 10.1111/j.1432-1033.1994.tb18947.x, PMID: 8026507

[40] Scanu A Luisetto R Oliviero F Gruaz L Sfriso P Burger D. High-density lipoproteins inhibit urate crystal-induced inflammation in mice. Ann Rheum Dis. (2015) 74:587–94. doi: 10.1136/annrheumdis-2013-203803, PMID: 24326007

[41] Dai C Yao X Keeran KJ Zywicke GJ Qu X Yu ZX. Apolipoprotein A-I attenuates ovalbumin-induced neutrophilic airway inflammation via a granulocyte colony-stimulating factor-dependent mechanism. Am J Respir Cell Mol Biol. (2012) 47:186–95. doi: 10.1165/rcmb.2011-0322OC, PMID: 22427535 PMC3423466

[42] Eyles JL Roberts AW Metcalf D Wicks IP. Granulocyte colony-stimulating factor and neutrophils--forgotten mediators of inflammatory disease. Nat Clin Pract Rheumatol. (2006) 2:500–10. doi: 10.1038/ncprheum0291 16951705

[43] Barugh AJ Gray P Shenkin SD MacLullich AM Mead GE. Cortisol levels and the severity and outcomes of acute stroke: a systematic review. J Neurol. (2014) 261:533–45. doi: 10.1007/s00415-013-7231-5, PMID: 24477489 PMC4928702

[44] Zhu F Ji Y Song JH Huang GX Zhang YF. Correlations between NLR, NHR, and clinicopathological characteristics, and prognosis of acute ischemic stroke. Medicine. (2023) 102:e33957. doi: 10.1097/MD.0000000000033957, PMID: 37327299 PMC10270530

[45] Zhao N Li J Zhang QX Yang L Zhang LJ. Elevated neutrophil-related immune-inflammatory biomarkers in acute anterior choroidal artery territory infarction with early progression. Clin Neurol Neurosurg. (2023) 229:107720. doi: 10.1016/j.clineuro.2023.107720, PMID: 37084652

